# Peritoneal dialysis-related peritonitis caused by *Fusarium*: a case report and literature review

**DOI:** 10.3389/ffunb.2025.1637498

**Published:** 2025-09-16

**Authors:** Qin Peng, Wenfeng Wu, Lirong Deng, Huanyue Tong, Huiyi Wu

**Affiliations:** ^1^ Clinical Pharmacy Center, Nanfang Hospital, Southern Medical University, Guangzhou, China; ^2^ Department of Clinical Pharmacy, The Second Clinical Medical College of North Sichuan Medical College, Nanchong, China

**Keywords:** *Fusarium*, peritonitis, peritoneal dialysis, voriconazole, therapeutic drug monitoring

## Abstract

Fungal peritonitis represents a significant complication of peritoneal dialysis (PD) and can result in severe consequences. However, fungal peritonitis caused by *Fusarium* is relatively rare, and there is no standard treatment plan for reference. Consequently, clinical pharmacists participated in a drug therapy for a rare case of fungal peritonitis in PD caused by *Fusarium* through literature review and therapeutic drug monitoring. Finally, this case received oral voriconazole, and the plasma concentration was maintained above 2 μg/ml. Moreover, the patient achieved favorable outcomes.

## Introduction

Peritonitis represents a significant complication of peritoneal dialysis (PD) and can result in severe consequences, including hospitalization, PD catheter extraction, and the necessity for permanent hemodialysis (Htay et al., 2018). The common pathogens of PD-associated peritonitis are predominantly Gram-positive and Gram-negative bacteria (Kim et al., 2004; Whitty et al., 2017). Conversely, fungal infections, particularly those caused by *Fusarium* spp., are infrequent (Hu et al., 2019; Kanjanabuch et al., 2022). In this study, we report on a case of the use of oral voriconazole for the treatment of PD-associated *Fusarium* peritonitis.

## Case report

A 61-year-old woman weighing 38.1 kg, who works as a farmer and had been undergoing continuous ambulatory peritoneal dialysis (CAPD) for over 8 years, presented with a 5-day history of abdominal pain, diarrhea, and vomiting, and then gradually developed cloudy PD effluent. Laboratory findings revealed a white blood cell (WBC) count of 12.6 × 10^9^/L, a neutrophil (NEU) count of 11.07 × 10^9^/L, and NEU% of 87.5%. Her C-reactive protein (CRP) level was 102.5 mg/L. Analysis of the PD effluent demonstrated turbidity with a WBC count of 4,044/μl (90% NEU). The admission diagnosis was PD-related peritonitis and chronic kidney disease (CKD 5).

Upon admission, empirical treatment with intraperitoneal (IP) cefazolin 0.5 g and amikacin 0.025 g four times daily was initiated. After 3 days, her laboratory tests showed a WBC count of 16.5 × 10^9^/L, NEU% of 94.5%, CRP level of 75.6 mg/L, and procalcitonin (PCT) level of 1.4 ng/ml. Analysis of the PD effluent showed turbidity with a WBC count of 4,210/μl (92% NEU). Due to inadequate response, the anti-infection regimen was adjusted to teicoplanin 0.02 g and meropenem 0.25 g (IP).

On day 7, the patient reported no improvement. Laboratory tests indicated a WBC count of 28.3 × 10^9^/L and NEU% of 93.8%. Her CRP was 131 mg/L and PCT was 2.1 ng/ml. Examination of the PD effluent revealed turbidity with a WBC count of 3,260/μl (91% NEU). Both the blood and PD effluent cultures yielded negative results. In light of the unresponsive nature of the treatment, the abdominal dialysis catheter was removed and changed to hemodialysis. Intravenous administration of meropenem and teicoplanin was initiated.

On day 13, with persistent elevation of the infection markers (i.e., CRP and PCT) and suspicion of fungal peritonitis, oral fluconazole 200 mg once daily was initiated and meropenem was discontinued. On day 23, the patient’s WBC count was 13.1 × 10^9^/L, NEU% was 84.1%, CRP was 103 mg/L, and PCT was 1.5 ng/ml. Culture of the PD effluent confirmed the presence of *Fusarium* spp. by microscopic examination. As a control, the culture of the PD fluid was negative. Teicoplanin and fluconazole were discontinued, and oral voriconazole was commenced with a loading dose of 200 mg on the first day, followed by a maintenance dose of 100 mg every 12 h. After 3 days, given a plasma concentration of voriconazole at 1.38 μg/ml by high-performance liquid chromatography, the maintenance dose was increased to 150 mg in the morning.

**Figure 1:**
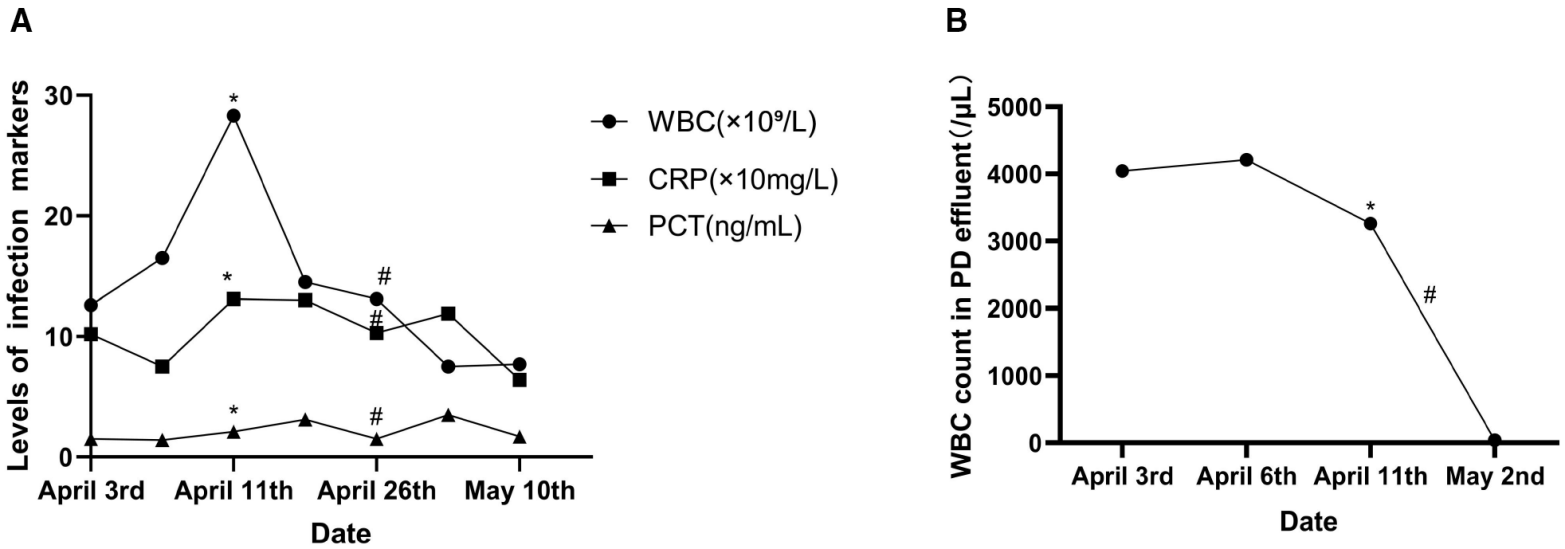
Changes in the infection markers. **(A)** Serum levels of the infection markers. **(B)** White blood cell (WBC) count in the peritoneal dialysis (PD) effluent. Asterisk represents removal of the PD catheter and the start of intravenous antimicrobial therapy. Number symbol denotes use of voriconazole. CRP, C-reactive protein; PCT, procalcitonin.

On day 37, the patient experienced resolution of abdominal pain. Laboratory analysis revealed a WBC count of 7.79 × 10^9^/L, NEU% of 80.7%, CRP of 64 mg/L, and PCT of 1.7 ng/ml. The plasma concentration of voriconazole measured 2.02 μg/ml. Upon discharge, the patient was prescribed voriconazole at doses of 150 mg in the morning and 100 mg in the evening. At the 2-month post-discharge evaluation, the patient remained afebrile and asymptomatic, with complete normalization of the inflammatory indices, indicating full clinical remission. The alterations in the medication and the key indicators throughout the hospital stay are depicted in **Figure 1**.

## Discussion

*Fusarium* spp. are filamentous fungi that produce mitospores and are ubiquitously present in the environment, inhabiting the air, water, and soil. They are recognized as significant plant pathogens. In humans, they act as opportunistic pathogens, causing localized or disseminated infection after trauma or weakened immunity. The common clinical pathogenic *Fusarium* species include *Fusarium solani*, *Fusarium verticillioides*, *Fusarium oxysporum*, and *Fusarium proliferatum*. Among these, *F. solani* stands out as the most prevalent and virulent species, accounting for approximately 40%–60% of infections. Management of *Fusarium* infections poses a challenge due to their multidrug resistance and their ability to cause a range of clinical presentations, such as pneumonia, fungemia, cellulitis, and lymphangitis, following skin trauma (Ledoux et al., 2024; Nucci and Anaissie, 2023). Despite these complexities, PD-related *Fusarium* peritonitis is a rare occurrence, and the available literature on this topic is limited. The *Fusarium* spp. infection in this case was likely related to her occupation as a farmer and her living environment. Moreover, as a patient with CKD 5 undergoing PD, she is not only repeatedly exposed to this environment during dialysis but is also immunocompromised, further increasing the risk of infection. Regrettably, neither the infecting subspecies nor the antimicrobial susceptibility profile could be determined.

In a multicenter study involving 88 patients with *Fusarium* infection, an analysis was conducted on the correlation between the minimum inhibitory concentration (MIC) of antifungal agents and the treatment outcomes. The mean MIC_50_ values of voriconazole against *F. solani* and *F. oxysporum* were determined to be 8 and 4 μg/ml, respectively, while those of amphotericin B were 2 and 1 μg/ml, respectively. In a comparative analysis, it was observed that there was no statistically significant variance in the mortality rates between voriconazole and amphotericin B liposomes. Conversely, amphotericin B deoxycholate exhibited a notably elevated mortality rate of 60% (Nucci et al., 2021). In addition, a retrospective multicenter investigation that included 233 instances of invasive *Fusarium* infection revealed similar 90-day survival rates for voriconazole and amphotericin B liposomes, while amphotericin B deoxycholate demonstrated a distinctly inferior outcome (Nucci et al., 2014). The 2021 Global Guidelines robustly advocate intravenous voriconazole or amphotericin B liposomes, either as monotherapy or in combination, as the primary therapeutic approach. Isavuconazonium or posaconazole is recommended as a second-line treatment. Notably, amphotericin B deoxycholate is not recommended in this context (Hoenigl et al., 2021).

At present, there exists a lack of consensus regarding the optimal management of peritonitis related to *Fusarium* spp. in patients with PD. **Table 1** displays a compilation and summary of the reported cases of *Fusarium* peritonitis from the references (Bibashi et al., 2002; da Silva-Rocha et al., 2015; Flynn et al., 1996; Garbino et al., 2005; García-Tapia et al., 1999; Gaur et al., 2010; Kerr et al., 1983; Liu and Sun, 2023; Rippon et al., 1988; Shah et al., 2014; Unal et al., 2011; Zhao et al., 2022). For drug selection, liposomal amphotericin B was unavailable, and amphotericin B deoxycholate was excluded due to its pronounced toxicity and poor prognosis. Intravenous voriconazole was withheld to avoid sulfobutyl-ether-β-cyclodextrin accumulation in CKD. Oral voriconazole, with equivalent bioavailability, was used instead and attained effective concentrations. Clinical pharmacists recommended the initial regimen of oral voriconazole for this patient, with a measured plasma concentration of 1.05 μg/ml. However, it is noteworthy that the MIC for *Fusarium* is relatively elevated. Studies have indicated that the MIC of the commonly obtained voriconazole is at least 2 μg/ml (Espinel-Ingroff et al., 2016), with a maximum of 5 μg/ml (Chen et al., 2018). Therefore, clinical pharmacists recommended an escalation in the voriconazole dosage to 150 mg in the morning. Subsequent monitoring revealed an increase in the plasma concentration to 2.02 μg/ml. This adjusted treatment regimen resulted in a notable alleviation of the patient’s abdominal pain and a substantial decrease in the infection markers.

**Table 1:** Cases of *Fusarium* peritonitis associated with peritoneal dialysis (PD).

Case	Pathogen	MIC (µg/ml)	Treatment	Serum concentration	Dialysis catheter	Prognosis
Case 13 (this article)	*Fusarium* spp.	None	VCZ: 150 mg (morning) and 100 mg PO (night)	VCZ: 2.02	Removed	Recovery
Case 1 (Kerr et al., 1983)	*Fusarium* spp.	None	AMB: 20 mg IV	None	Removed	Recovery
Case 2 (Rippon et al., 1988)	*Fusarium verticillioides*	None	AMB: 30 mg/day IV, three times 30 mg IV, 2/weekly Total dose: 1.5 g	None	Removed	Recovery
Case 3 (Flynn et al., 1996)	*Fusarium* spp.	None	AMB: 0.5 mg kg^−1^ day^−1^ IV (23 days; total dose, 79.4 mg) 5.0 mg/L IP (2 days)	None	Removed	Recovery
Case 4 (García-Tapia et al., 1999)	*Fusarium oxysporum*	AMB = 1 KCZ = 0.125 FCZ = 0.5 ITZ = 0.06	AMB: 50 mg/day IV, 2 weeks	None	Removed	Recovery
Case 5 (Bibashi et al., 2002)	*Fusarium solani*	None	AMB: 0.3 mg kg^−1^ day^−1^ IV, 4 weeks KCZ: 200 mg/day, 10 days	None	Removed	Recovery
Case 6 (Garbino et al., 2005)	*Fusarium* spp.	VCZ, sensitive. AMB, FCZ, and ITZ, resistance	VCZ: 6 mg/kg, q12h, day 1 4 mg/kg IV, q12h, 2 months	None	Removed	Recovery
Case 7 (Unal et al., 2011)	*Fusarium* spp.	None	AMB	None	Removed	Recovery
Case 8 (Gaur et al., 2010)	*Fusarium dimerum*	AMB = 1 VCZ = 2 ITZ = 16	AmBL: 3 mg kg^−1^ day^−1^ IV, 2 months	None	Removed	Recovery
Case 9 (Shah et al., 2014)	*Fusarium solani*	AMB = 4 POS = 8 VCZ = 8 ITZ = 16	POS: 800 mg/day, then change for 600 mg/day, 3 months	None	Removed	Recovery
Case 10 (da Silva-Rocha et al., 2015)	*Fusarium solani*	FCZ = 64 ITZ = 16 MCF = 8 AMB = 1	AMB: 50 mg/day, 4 weeks	None	Removed	Recovery
Case 11 (Zhao et al., 2022)	*Fusarium solani*	AMB = 0.5 VCZ = 3 ITZ > 32	VCZ inefficacious (10 days) change for POS 400 mg bid, 12 weeks	VCZ: 3.7–4.5	Removed	Recovery
Case 12 (Liu and Sun, 2023)	*Fusarium verticillioides*	None	VCZ+AMB: 20 mg/72 h (total dose, 1.5 g)	None	Removed	Recovery

*MIC*, minimum inhibitory concentration; *AMB*, amphotericin B; *AmBL* liposomal amphotericin B; *FCZ*, fluconazole; *VCZ*, voriconazole; *ITZ*, itraconazole; *KCZ*, ketoconazole; *POS*, posaconazole.

## Conclusion

PD-related peritonitis caused by *Fusarium* spp. is a rare occurrence. The diagnosis was confirmed for this case due to meeting all three of three diagnostic criteria: abdominal pain and cloudy effluent; effluent WBC count >100 × 10^6^/L (4,460 × 10^6^/L) with >90% NEU; and effluent culture positive for *Fusarium* spp. However, the pathogen was not cultured until 13 days after admission, which delayed the treatment to some extent. Rapid assays such as the (1→3)-β-d-glucan test, the galactomannan test, and next-generation sequencing of the blood or peritoneal dialysate samples can accelerate the identification of rare fungal peritonitis.

Large-scale investigations focusing on antifungal interventions for *Fusarium* peritonitis are scarce, with the current therapeutic strategies predominantly documented through isolated case reports. A key limitation is the absence of *Fusarium* susceptibility data, leaving antifungal selection empirical rather than targeted. Fortunately, in this case, clinical pharmacists conducted a comprehensive review of the pertinent literature, evaluated the epidemiological aspects of *Fusarium* infections, appraised the pharmacological profiles of therapeutic agents, and collaborated with clinicians to devise personalized antifungal regimens incorporating therapeutic drug monitoring. Finally, despite the eventual recovery, the patient and her family lamented the delayed identification of the pathogen that needlessly prolonged hospitalization. This case underscores the significance of oral voriconazole combined with therapeutic drug monitoring in the management of peritonitis associated with *Fusarium* in PD and serves as a valuable reference regarding its efficacy.

## Data Availability

The original contributions presented in the study are included in the article/supplementary material. Further inquiries can be directed to the corresponding author.
